# Primary hepatic extranodal marginal zone B-cell mucosa-associated lymphoid tissue lymphoma treated by laparoscopic partial hepatectomy: a case report

**DOI:** 10.1186/s40792-023-01613-y

**Published:** 2023-02-27

**Authors:** Keisuke Okura, Satoru Seo, Hironori Shimizu, Hiroto Nishino, Tomoaki Yoh, Ken Fukumitsu, Takamichi Ishii, Koichiro Hata, Hironori Haga, Etsuro Hatano

**Affiliations:** 1 Department of Surgery, Graduate School of Medicine, Kyoto University, Kyoto, Japan; 2 Department of Surgery, Kyoto Katsura Hospital, 17 Yamadahirao-cho, Nishikyo-ku, Kyoto, 615-8256 Japan; 3 Department of Diagnostic Imaging and Nuclear Medicine, Graduate School of Medicine, Kyoto University, Kyoto, Japan; 4 Department of Diagnostic Pathology, Graduate School of Medicine, Kyoto University, Kyoto, Japan

**Keywords:** Hepatectomy, Laparoscopy, MALT lymphoma

## Abstract

**Background:**

Primary hepatic extranodal marginal zone B-cell mucosa-associated lymphoid tissue (MALT) lymphoma is very rare, so it is difficult to diagnose preoperatively. And there is no established treatment for hepatic MALT lymphoma. We report herein a case of primary hepatic MALT lymphoma treated by laparoscopic partial hepatectomy, and discuss the usefulness of laparoscopic hepatectomy for a rare liver tumor.

**Case presentation:**

This patient was a woman in her 60s, who was diagnosed preoperatively as having synchronous liver metastasis from sigmoid colon cancer; therefore, laparoscopic partial hepatectomy was performed. She had a good course after the operation and was discharged on postoperative day 12. However, she was diagnosed pathologically as having primary hepatic MALT lymphoma. A bone marrow biopsy was also performed, and then she was finally diagnosed as having limited-stage primary hepatic MALT lymphoma. She received no postoperative treatment and showed no recurrence for 4 years postoperatively.

**Conclusions:**

We experienced the good result of the patient with limited-stage primary MALT lymphoma treated by laparoscopic partial hepatectomy. Liver tumors are sometimes misdiagnosed by imaging examinations alone. Laparoscopic hepatectomy has been widespread recently as a minimally invasive procedure, and it may be useful for both diagnosis and treatment.

## Background

One of the indolent lymphomas is an extranodal marginal zone B-cell mucosa-associated lymphoid tissue (MALT) lymphoma. MALT lymphoma occurs most frequently in the stomach [1], but it can occur in various areas. In particular, primary liver MALT lymphoma is quite rare [2].

This report presents a case of primary hepatic MALT lymphoma treated by laparoscopic partial hepatectomy. The patient had been initially diagnosed as having synchronous liver metastasis from sigmoid colon cancer, but the pathological diagnosis was primary hepatic MALT lymphoma postoperatively. In this case, it was not until the resection of the liver tumor that it was diagnosed, and the treatment was completed only with laparoscopic hepatectomy, which was minimally invasive.

## Case presentation

**Fig. 1:**
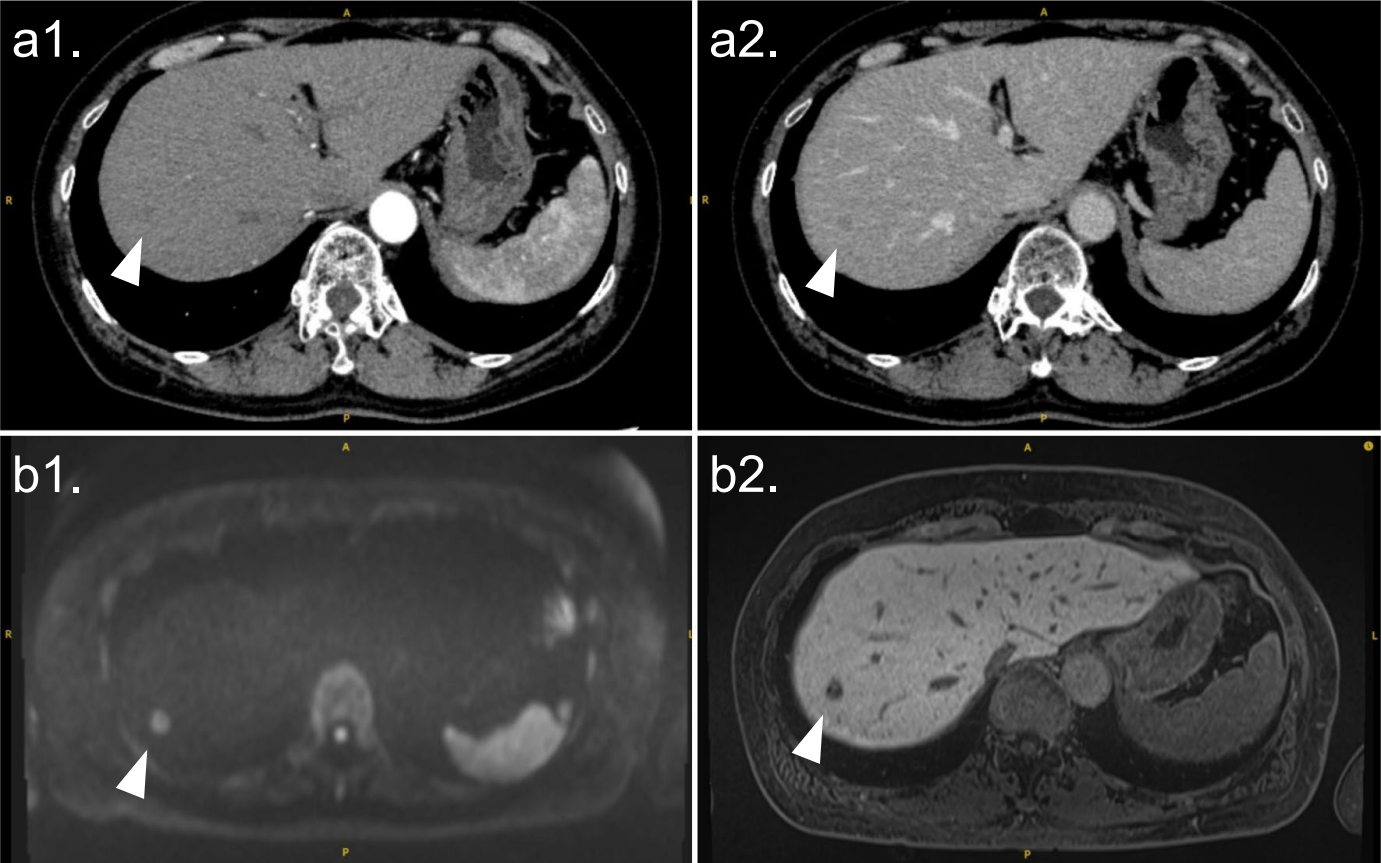
Preoperative imaging examinations. MDCT shows a low-density nodule, about 1 cm in diameter during the arterial phase (**a1**) and ring enhancement during the delayed phase (**a2**) in liver segment 8. EOB-MRI shows restricted diffusion (**b1**) and a low-signal intensity mass during the hepatobiliary phase (**b2**) in liver segment 8. *MDCT* multidetector computed tomography, *EOB-MRI* Gd-EOB-DTPA-enhanced magnetic resonance imaging.

The patient was a woman in her 60s. She visited a nearby hospital because of bloody stool. She underwent a careful examination, and she was diagnosed as having sigmoid colon cancer with synchronous liver metastasis. She underwent laparoscopic sigmoid colectomy, and the pathological diagnosis based on the Japanese classification was as follows: S, Type2, 20 × 20 mm, moderately differentiated tubular adenocarcinoma (tub2), pT3(SS), med, INFb, ly0, v1, pN0(0/16), EX/ND(-), PN1a, pPM0 (90 mm), pDM0 (50 mm), pRM0. She received postoperative systemic chemotherapy. Then, no new lesion occurred in the liver. She preferred to undergo laparoscopic hepatectomy and was referred to our hospital. She tested negative for serum marker of hepatitis B and C virus infection. Additionally, several types of imaging examinations were performed: multidetector computed tomography (MDCT), Gd-EOB-DTPA-enhanced magnetic resonance imaging (EOB-MRI), and ^18^F-fluorodeoxyglucose-positron emission tomography (FDG-PET). MDCT showed a low-density nodule during the arterial phase (Fig. 1a1) and ring enhancement during the delayed phase in liver segment 8 (Fig. 1a2). EOB-MRI showed restricted diffusion (Fig. 1b1) and a low-signal intensity mass during the hepatobiliary phase in liver segment 8 (Fig. 1b2). However, FDG-PET showed no marked uptake in the liver. She was diagnosed as having a liver metastasis from sigmoid colon cancer as initially diagnosed, and laparoscopic partial hepatectomy was planned.

**Fig. 2:**
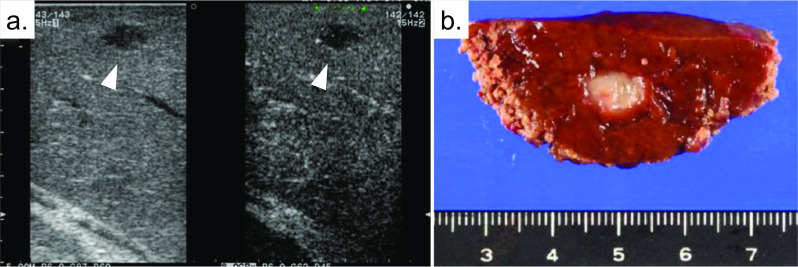
Operative findings**.** **a** Contrast-enhanced ultrasonography with perfluorobutane microbubbles during the operation, and **b** the resected specimen of liver segment 8

The trocar was placed, with no adhesions and no ascites in the abdominal cavity. The liver was examined intraoperatively by contrast-enhanced ultrasonography with perfluorobutane microbubbles (Fig. 2a), and then laparoscopic partial resection of liver segment 8 was performed with the Pringle maneuver. The cross-section of the resected specimen is shown in Fig. 2b. During the operation, there were no complications, and there was a small amount of blood loss.

The patient had a good postoperative course, and she was discharged on postoperative day 12.

**Fig. 3:**
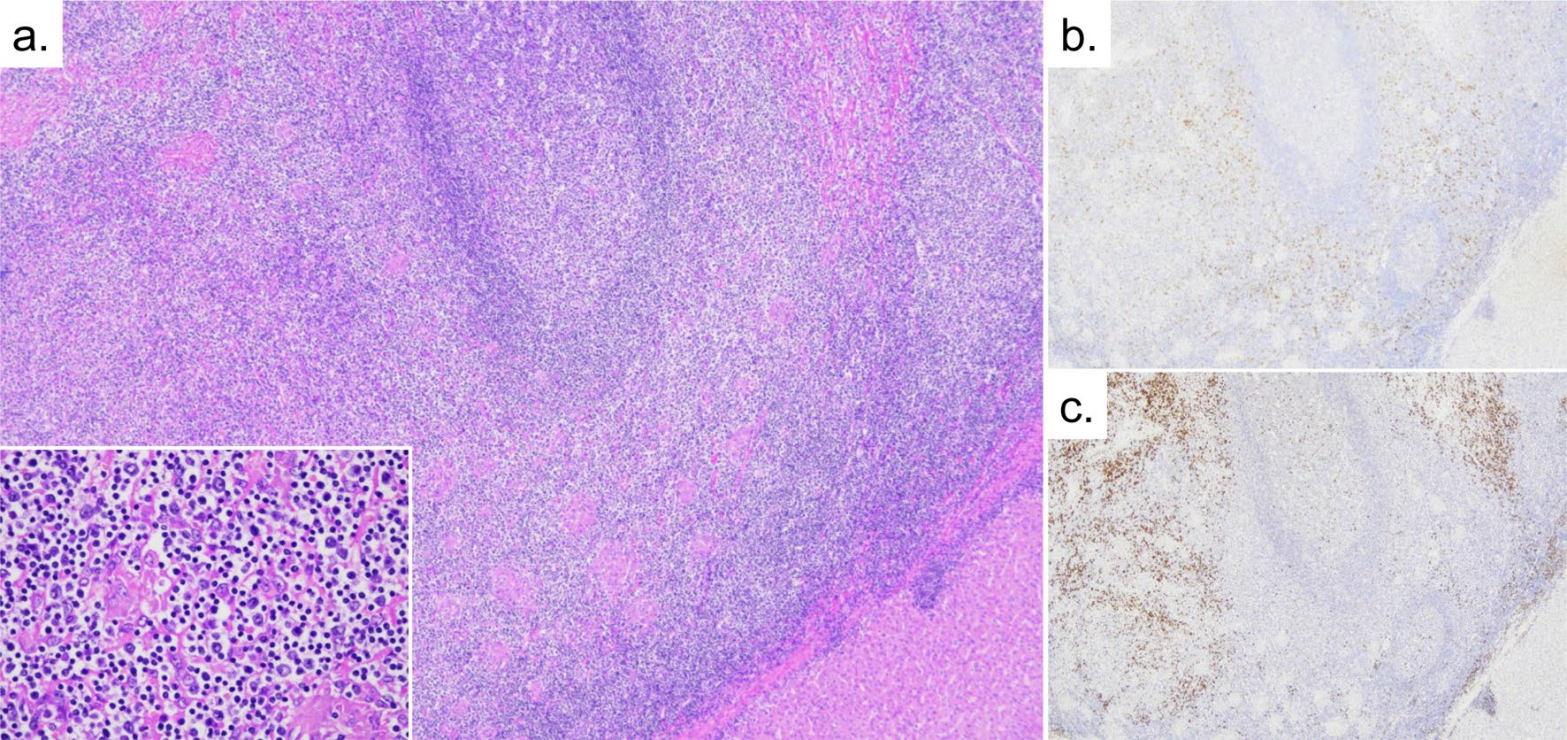
Pathological findings of the resected specimen. Low-power view shows nodular lymphoplasmacytic proliferation containing lymph follicles, and the high-power view shows monocytoid B cell proliferation. **a** Hematoxylin and eosin staining (low and high-power view), **b** IRTA1 (FCRL4) (low-power view), **c** kappa chains (low-power view)

The pathological findings were as follows. The nodule in the liver parenchyma was composed of dense lymphoplasmacytic proliferation, and scattered lymph follicles were surrounded by intermediate-sized B cells with clear cytoplasm, suggesting marginal zone/monocytoid B cell differentiation (Fig. 3a). Those cells were positive for CD20 and IRTA1(FCRL4) (Fig. 3b) and negative for CD3 and CD10. Some areas showed aggregates of plasmacytic cells, which were predominantly positive for kappa chains (Fig. 3c). Based on the above, the tumor cells were positive for CD20, IRTA1, and kappa chains, and negative for CK20, CD3, CD10, and lambda chains. The Ki-67 labeling index was 20%. These findings are consistent with a diagnosis of extranodal marginal zone B-cell lymphoma of mucosa-associated lymphoid tissue. Based on these pathological findings, the diagnosis was hepatic MALT lymphoma, and a hematologist was consulted. He performed a bone marrow biopsy. She was finally diagnosed as having limited-stage primary hepatic MALT lymphoma. She did not receive any treatment after the operation, and she has shown no recurrence for 4 years.

## Discussion

Primary hepatic MALT lymphoma is quite rare and was first reported by Peter G. Isaacson in 1995 [3]. It accounted for only 3% of 180 cases of extragastric MALT lymphoma in the previous study [2]. The present patient was diagnosed preoperatively as having synchronous liver metastasis from sigmoid colon cancer. However, the postoperative pathological diagnosis was primary hepatic MALT lymphoma. She was misdiagnosed preoperatively due to the rarity of primary hepatic MALT lymphoma and the lack of imaging findings specific for hepatic MALT lymphoma.

Liver tumors are diagnosed mainly by imaging examinations. At our hospital, we usually perform MDCT, EOB-MRI, and FDG-PET. This case showed ring enhancement during the delayed phase on MDCT and restricted diffusion on EOB-MRI. However, it did not show arterial phase enhancement or the vessel penetration sign. From the above findings, liver metastasis from sigmoid colon cancer or primary intrahepatic cholangiocarcinoma was diagnosed, and, therefore, surgical resection was performed. Primary hepatic MALT lymphomas were reported to be characterized by arterial phase enhancement, restricted diffusion, the vessel penetration sign, and, more specifically, “speckled enhancement” in the hepatobiliary phase of EOB-MRI. “Speckled enhancement” refers to punctate positive enhancement within the low-signal intensity lesions on the hepatobiliary phase of EOB-MRI [4]. However, even when looking back at this case, the imaging examination showed no specific findings of primary hepatic MALT lymphoma, and it was difficult to make a precise diagnosis before resection in this case.

Treatment for MALT lymphoma depends on two main aspects: the primary involved organ and the extension of disease [5]. Gastric MALT lymphoma is associated with *Helicobacter pylori*, so *Helicobacter pylori* eradication therapy should be given to all patients with gastric MALT lymphoma, irrespective of stage [6]. There was a previous report that, for extragastric MALT lymphoma, radiation, surgery, and chemo/immunotherapy seemed to be equally effective in achieving remission and prolonged progression-free survivals, but their curative potential is questionable [7], because extragastric MALT lymphoma showed multiorgan involvement more often than gastric MALT lymphoma [8]. In the present case, the liver tumor was judged to be resectable, and laparoscopic partial hepatectomy was performed. A bone marrow biopsy was performed postoperatively, and she was finally diagnosed as having limited-stage primary hepatic MALT lymphoma. She showed no recurrence for 4 years without postoperative treatment, and long-term follow-up is recommended due to the potential life-long risk of recurrence [5]. On the other hand, there are some case reports of hepatic MALT lymphoma treated by radiofrequency ablation (RFA) [9, 10]. If the liver tumor is small, it may be an indication for RFA, but it cannot be diagnosed correctly without a biopsy. It is careful in which case we should treat for RFA.

In the present case, laparoscopic partial hepatectomy was performed unexpectedly for primary hepatic MALT lymphoma. There are only a few case reports of pure laparoscopic hepatectomy for primary hepatic MALT lymphoma [11–14] (Table 1). Three patients were misdiagnosed preoperatively, as in the present case, and the other case report was not described about the diagnosis. We summarized the characteristics of the preoperative images in 5 patients treated by laparoscopic resection (Table2). Due to the improvement of diagnostic images, each size of these tumors was so small that there is no consistent trend about the image findings. For the literature review, we searched PubMed, using the key words, which included “primary hepatic MALT lymphoma” and “surgery”, “resection” or “hepatectomy”. To our best knowledge, there are 25 English case reports including 27 patients [3, 15–38] (Table 3). According to our literature review, this study consists of 17 males and 10 females with a median age of 60 years. Ten patients underwent liver biopsy preoperatively, but it was still difficult to diagnose some patients correctly. Some liver tumors were found incidentally in 2 patients during the operation for the other disease. If the diagnosis of liver tumors needs to be confirmed preoperatively, liver biopsy is often useful. However, we should take into account the disadvantage of dissemination when conducting liver biopsy. Recently, laparoscopic hepatectomy for liver tumors has been widely conducted; it is easier to safely resect them than previously. Laparoscopic hepatectomy may be useful for both diagnosis and treatment.

**Table 1:** Previous case reports about primary hepatic MALT lymphoma treated by laparoscopic hepatectomy

Case	Age	Sex	Size (cm)	Preoperative diagnosis	Procedure	Extrahepatic lesion	Outcome
Fujiwara [11]	71	Female	1.5	Cholangiocarcinoma	Lap partial hepatectomy	None	NED, 3 years
Khurana [12]	71	Female	1.5 × 1	N.D	Lap partial hepatectomy	N.D	N.D
Xie [13]	73	Male	1.8	Hepatocellular carcinoma	Lap left lateral sectionectomy	N.D	NED, 6 months
Fu [14]	58	Female	1.0 × 0.8, 0.8 × 0.4	Hepatocellular carcinoma	Lap left lateral sectionectomy	None	NED, 17 months
Our case	60s	Female	1.0	Liver metastasis from sigmoid colon cancer	Lap partial hepatectomy	None	NED, 4 years

*Lap* laparoscopic, *N.D* not described, *NED* no evidence of disease

**Table 2:** The characteristics of the preoperative image findings in the previous case reports about primary hepatic MALT lymphoma treated by laparoscopic hepatectomy

Case	Size (cm)	Vascularity			Preoperative diagnosis
		Arterial phase	Portal venous phase	Delayed phase	
Fujiwara [11]	1.5	Hypo*	Hypo*	Hypo*	Cholangiocarcinoma
Khurana [12]	1.5	N.D	N.D	N.D	N.D
Xie [13]	1.8	Slight hyper*	N.D	N.D	Hepatocellular carcinoma
Fu [14]	1.0 × 0.8	Hyper†	Hyper†	Hypo†	Hepatocellular carcinoma
	0.8 × 0.4	Slight hyper†	Slight hyper†	Slight hyper†	
Our case	1.0	Hypo*	Ring enhancement*	Ring enhancement*	Liver metastasis from sigmoid colon cancer

*N.D* not described

*judged by CT, †judged by MRI

**Table 3:** Reported case reports of the patients with primary hepatic MALT lymphoma treated by laparotomy

Case (year)	Age	Sex	Number	Size (cm)*	Preoperative diagnosis	Liver biopsy before surgery	Postoperative adjuvant therapy	Outcome
Isaacson (1995)	66	Male	Solitary	7.5	Low-grade lymphoma	+		NED, 1 year
	73	Female	Solitary	3.0	(incidental)	−		N.D
Ueda (1996)	48	Male	Solitary	4.5	MALT lymphoma	+	Chemotherapy	NED, 3 years
Maes (1997)	47	Female	Solitary	4	Low-grade B-cell lymphoma	+	Radiotherapy	NED, 2.5 years
	64	Male	Solitary	2	Liver metastasis from colon cancer	−		N.D
Prabhu (1998)	62	Female	Solitary	6.0	Small lymphocytes	+		N.D
Chen (2000)	64	Female	Solitary	N.D	N.D	−		REC, 8 years
Mizuno (2002)	59	Male	Solitary	1.5	HCC	−		NED, 2.5 years
Murakami (2002)	61	Male	Solitary	3.4	Low-grade B-cell lymphoma	+		NED, 1.5 years
Yago (2002)	73	Male	Solitary	4	Non-Hodgkin lymphoma	+		NED, 34 months
Takeshima (2004)	65	Female	Multiple	2	Chronic hepatitis with lymphoid follicles	+		NED, 10 months
Gockel (2005)	36	Male	Solitary	6	MALT lymphoma	+		REC, 14 months
Doi (2008)	58	Male	Solitary	2.5	HCC	−	Chemotherapy	NED, 6 months
Koubaa Mahjoub (2008)	59	Male	Solitary	2	(incidental)	−		NED, 5 months
Yu (2013)	38	Male	Multiple	2.5	MALT lymphoma	+	Chemotherapy	NED, 15 months
Zhong (2014)	53	Male	Solitary	4.5	Malignant	−	Chemotherapy	NED, 40 months
Chan (2015)	59	Male	Solitary	2.5	N.D	−		NED, 4 years
Nagata (2015)	74	Male	Solitary	1.5	ICC	−		NED, 2 years
Shiozawa (2015)	60	Female	Solitary	1.5	Malignant	−		N.D
Li (2016)	44	Female	Solitary	1.8	HCC	−		NED, 27 months
Betianu (2017)	47	Female	Solitary	8.5	Benign	−	Chemotherapy	NED, 9 months
Dong (2017)	50	Male	Solitary	5	ICC	−	Chemotherapy	NED, 13 months
Obiorah (2017)	80	Female	Solitary	N.D	HCC	−		REC, 1 year
Bohlok (2018)	68	Male	Solitary	4.5	HCC	−		N.D
Choi (2020)	70	Male	Multiple	4.8	HCC	−		NED, 8 months
Yasuda (2020)	54	Female	Multiple	N.D	HCC or ICC	−		NED, 12 months
Liu (2022)	65	Male	Solitary	7	Lymphoid tissue hyperplasia with dysplasia	+		alive, 20 months

*HCC* hepatocellular carcinoma, *ICC* intrahepatic cholangiocarcinoma, *N.D* not describe, *NED* no evidence of disease, *REC* recurrence

*This indicates the maximum diameter of the tumor(s)

## Conclusions

Primary hepatic MALT lymphoma is a very rare disease, and it is often difficult to diagnose precisely without biopsy. However, the problem of liver biopsy is dissemination. Laparoscopic hepatectomy has been widespread recently as minimally invasive surgery, so laparoscopic hepatectomy should be considered for the treatment of a resectable liver tumor if there is confusion about the diagnosis.

## Data Availability

Not applicable.

## Abbreviations

MALT: Mucosa-associated lymphoid tissue
MDCT: Multidetector computed tomography
EOB-MRI: Gd-EOB-DTPA-enhanced magnetic resonance imaging
FDG-PET: ^18^Fluorodeoxyglucose-positron emission tomography
RFA: Radiofrequency ablation
